# Cross-Sectional Descriptive Pilot Study on the Risk of Psychotic Disorders among Adolescents

**DOI:** 10.3390/children8100916

**Published:** 2021-10-14

**Authors:** Carmen Cendrero-Luengo, María Jiménez-Palomares, Juan Rodríguez-Mansilla, Elisa María Garrido-Ardila

**Affiliations:** 1 Faculty of Medicine, San Joan d’Alacant Campus, Miguel Hernández University, 03550 San Joan d’Alacant, Spain; carmen.cendrero@goumh.umh.es; 2 ADOLOR Research Group, Department of Medical-Surgical Therapy, Faculty of Medicine and Health Sciences, Extremadura University, 06006 Badajoz, Spain; mariajp@unex.es (M.J.-P.); egarridoa@unex.es (E.M.G.-A.)

**Keywords:** psychotic disorder, adolescence, risk factors, early intervention, occupational therapy

## Abstract

Background: Adolescence is a key stage for the development of different mental disorders, particularly psychotic disorders. This stage of life is accompanied by new habits or behaviours that can make a person more vulnerable to developing a psychotic disorder or, on the contrary, play a protective role. Objective: To study the vulnerability to developing a psychotic disorder in adolescents and to analyse the main risk factors. Materials and methods: This is an observational, descriptive and cross-sectional study. The data collection was conducted using the Prodromal Questionnaire Brief Version (PQ-B) test and a self-developed questionnaire based on the vulnerability–stress model. Results: Of the total sample (*n* = 44), 65.9% were male and 100% lived in a rural environment. In general, among risk factors (stress, alcohol and cannabis consumption, history, bullying, traumatic event and sedentary lifestyle), alcohol consumption and the presence of stress were found to have high values. Furthermore, a risk of psychosis was found in 38.6% of the studied population. Of this percentage of adolescents at risk, 64.7% consumed alcohol (*p* = 0.99) and 82.4% suffered from stress (*p* = 0.7161). The protective factor (physical activity) was found in more than half (59% *p* = 0.16). Conclusions: There is a high risk of psychosis among the young adolescents assessed in this study, where the explanatory factors identified with higher values were alcohol consumption and the presence of stress.

## 1. Introduction

Psychotic disorders are severe mental disorders, which are defined by the current edition of the Diagnostic and Statistical Manual of Mental Disorders (DSM-V) [1] as syndromes rather than diseases. They are listed under the category ‘Schizophrenia spectrum and other psychotic disorders’. This category highlights that psychotic disorders are schizophrenia, other psychotic disorders and schizotypal (personality) disorder. Each psychotic disorder has a specific diagnosis and is distinguished mainly by its duration. However, what brings them all into the same category is the presence of abnormalities in one or more of the following domains: delusions, hallucinations, disorganised thinking, grossly disorganised or abnormal motor behaviour, and negative symptoms [1,2].

The prognosis of psychotic disorders worsens with late detection of symptoms. Therefore, the early detection of clinical manifestations is very important [1]. Attention to the prodromal phases of these disorders has only been recently established. Since then, management strategies have focused on early detection for the prescription of the most appropriate treatment approach [3]. The psychosis continuum includes a variety of psychotic symptoms that can be observed from the subclinical stages to the diagnosis and posterior stages of a psychiatric disorder, this is to say from the subclinical symptoms to the clinically significant symptoms [4]. In relation to this range of symptoms, there is controversy as to whether the psychotic symptoms are in a continuum with the psychoticlike experiences that healthy individuals can have. However, the scientific evidence indicates that the risk of developing a psychotic disorder increases in persons that have had psychoticlike experiences and have been exposed to risk factors [5]. The psychosis continuum model also supports that the transition individuals make through this spectrum throughout life will be determined by the protective factors and risk factors of the subject, and those determined by their family and social environment. Thus, strengthening the protective factors (promotion) and minimising the risk factors (prevention) can enable the individual to have adequate mental health [6]. Adolescence is a key stage for implementing this early detection, as it is a phase of life in which the person has to face neurophysiological, physical, cognitive, emotional and social changes induced by a more active cerebral cortex [2,3,7]. Poor mental health in adolescence can have a direct impact on the development of the person, limiting the potential of the adolescent. It can also have negative consequences, such as impaired social function, low educational attainment, unemployment, substance abuse, violence and suicide [3]. Currently, the literature on the factors that may affect the mental health of adolescents is very heterogeneous but the evidence indicates that stress, bullying and the consumption of toxic substances are the main explanatory factors [8,9]. Adolescence is a stage of life when the cerebral cortex is very active and therefore susceptible to irreversible and scattered changes, which may be magnified by these exogenous factors induced by new habits [8,9]. Therefore, it is sometimes difficult to discern what may trigger these changes [9]. Although the available scientific evidence is diverse and sometimes controversial, the available literature agrees on several aspects [9,10,11,12,13,14,15]. Some authors [8,9] warn that at least half of all mental disorders appear around the age of 14. Gender can be considered as another triggering factor, as psychotic disorders are more frequent in males than in females at younger ages and appear at an earlier age in males [2,10].

The habits and routines that are established at this stage of the life cycle are an essential trigger. The biological predisposition, together with practices such as alcohol and cannabis consumption, which begin at around 13–16 years of age, have an impact on the mental health of the adolescents [9]. Current evidence shows that high levels of cannabis intake increase the risk of psychotic disorders and confirms a dose–response relationship between the level of consumption and the risk of psychosis [11]. Additionally, there is a causal relationship between alcohol use and mental disorders [12]. Both substances are psychoactive and alter the neurophysiological brain dynamics [13,14,15].

Stress is considered both as a precipitating environmental factor and as a marker of a predisposing vulnerability to the development of a psychotic disorder [16,17]. The effect of stress could be responsible for the deregulation of the brain’ pathway mechanisms, modifying the biological pathways involved in psychotic disorders [17,18]. Another environmental factor related to the problem, which has a negative impact on the stress levels, is bullying [19]. Several studies relate bullying to a continuous exposure to stress where victims of bullying report some psychotic disorders [20,21,22,23]. Conversely, practices such as physical exercise may be a protective factor in influencing mental health [24], acting as a regulatory releaser of positive neurotransmitters and enhancing the activity of the cerebral cortex [25]. These factors play an important role in understanding the transition of an individual from a vulnerable state to the development and diagnosis of the psychotic disorder [13]. Currently, one of the models that contemplates and studies the interaction between exogenous factors and their influence on mental health is the vulnerability–stress model [26]. This explanatory model is a theoretical formulation of the origin of psychosis, which is based on a predisposition to psychosis together with a vulnerability triggered by exogenous factors, giving rise to this disorder [26]. As these are exogenous and controllable factors, it is also important to focus on these detrimental practices for the mental health of adolescents. In addition, it is essential to pay attention to the importance of the family environment, which can influence the adolescent in a positive or negative way [13]. The family lifestyle and routines can enhance these risk factors, encouraging habits that compromise mental health, or, on the contrary, act as early detectors of the psychotic disorders [10,11,13]. Therefore, raising awareness among families and the general population in relation to this problem is essential [2,8,9,10,11,19].

Regarding treatment, the management of the psychotic disorders was traditionally focused on the residual phase of the condition and consisted mainly in drug intake [27]. As treatment approaches have been changing and developing, the role of professions such as occupational therapists, which offer non-pharmacological treatment for these types of disorders, have also been evolving. In fact, this discipline is currently being used in early detection programmes. Ramón and Mainar [28] stated that the role of the occupational therapist in the prodromal phase of the psychotic disorders ‘is to minimise the impact of symptoms on functionality ensuring that, in the event that a psychotic break occurs, the patient has a maximum level of functionality, which is an indicator of a better prognosis’.

To date, the available research that analyses the extrinsic risk factors that contribute to the development of psychosis in adolescence has been mainly carried out in populations of Anglo-Saxon and Nordic countries [29]. Likewise, on the basis of our knowledge, there are no previous studies that assess the risk of developing a psychotic disorder in Spanish adolescents. Therefore, the present research is an exploratory study which constitutes a pioneering and novel study in the Spanish population and aims to carry out a first screening of the problem. In this sense, it is important to take into account that the research on contributing factors and vulnerability to developing a psychotic disorder could provide useful knowledge that will contribute to improving and implementing prevention programmes that include occupational therapy. This will prevent harmful and detrimental consequences for the health of adolescents, as well as minimise the appearance of irreversible problems in adulthood.

### Aim of the Study

The objective of this study was to analyse the risk of vulnerability to develop a psychotic disorder in adolescents between 14 and 18 years of age and to assess the risk of psychosis, as well as the risk factors that contribute to the development of this condition.

## 2. Materials and Methods

The present study is an observational, descriptive and cross-sectional study. The research was carried out from December 2018 to July 2019.

Participants: The target population were all the students of the ‘Virgen de Altagracia’ Secondary School in the town of Siruela (Badajoz, Extremadura region, Spain) who were 46 adolescents in total. The inclusion criteria established were: students within an age range from 14 to 18 years (both ages included), informed consent signed by their legal guardian and voluntary participation of the student. The exclusion criteria were the absence of a signed informed consent, not belonging to the aforementioned institute and having been diagnosed with any type of disability.

Ethical aspects: The study was approved by the Bioethics and Biosafety Committee of the University of Extremadura (registration number 176/2019). The legal guardians of the adolescents signed the Informed Consent for the participation of their children in the study. All the students that participated in the study agreed to collaborate in the research.

### 2.1. Measures

The primary outcome measure was the risk of psychosis. This variable was measured with the Prodromal Questionnaire Brief Version (PQ-B) in its Spanish version adapted and validated by Fonseca et al. [30] in Spanish adolescents [2], from its original version by author Lowey et al. published in 2011 [31]. The PQ-B is a brief, simple and reliable psychosis risk screening measure containing 21 items that are answered in a dichotomous (true/false) response format [31]. The PQ-B asks additional questions regarding the extent/severity of impairment and distress, scored in Likert-type (1 = Strongly Disagree to 5 = Strongly Agree) [2,31]. The Spanish adaptation of the BQP has been shown to have adequate psychometric properties [31] and it has been used in many studies for screening adolescents at risk of psychosis [32,33,34,35,36,37]. In terms of interpretation, according to the lead author [31], a score of 6 or higher indicates that the person is at risk of developing psychosis [30].

The secondary outcome measures were the sociodemographic features and explanatory factors (contributing and protective) of developing a psychotic disorder. An ad hoc questionnaire was used in order to obtain this information. It was designed and developed by the authors of the study based on the most recent literature on the study subject [9,10,11,12,13,14,15,16,27]. Databases such as Pubmed, Science Direct and Scopus were consulted. The following sociodemographic data were collected: age, gender, place of origin, cohabiting members of the family and economic level. The questionnaire also included different risk factors related to psychotic disorders (stress, substance use, age, sex, background and clinical history, bullying and traumatic event experienced). The questionnaire was developed on the basis of the vulnerability–stress model, which establishes the interaction between genetic factors, sociodemographic variables (gender, age) environmental factors (stress, substance use) and the risk of psychosis [26].

### 2.2. Procedure

A total of 46 students were identified as potential study participants and their parents/legal guardians were contacted. The parents were given an envelope with all the documents and information about the study. This included a participant information sheet with all the details of the project, a booklet with the questionnaires and the instructions on how to complete them and the informed consent. After explaining the study procedure to them, 44 parents/legal guardians signed the informed consent form and agreed to participate in the study. As the response rate was 95.6%, we proceed to the data collection. The two questionnaires were distributed at the secondary school in paper format to all the students included in the study. An explanation of the survey and the questions was provided to the participants before completing the questionnaires. Once the students completed the questionnaires, they were collected by one researcher and placed in a sealed envelope. Another researcher independent to the study was in charge of data collection from the questionnaires.

### 2.3. Data Analysis

The statistical analysis was carried out using the R 4.0.3 program (R Foundation for Statistical Computing, Vienna, Austria; http://www.R-project.org (accessed on 14 May 2021)). A descriptive analysis was performed presenting qualitative variables as *n* and percentage. Statistical significance was established at a value of *p* < 0.05. The characteristics of the study participants were described according to the risk of psychosis as a categorical variable. When the re-categorization of the variable was performed, the groups “at risk” and “not at risk” were included, according to the limit scores established by the PQ-B (>6 or < or =6, respectively). Additionally, the explanatory factors were described as categorical variables according to the risk of psychosis. The statistical inference tests used were the Fisher’s Exact test or the Chi-square test. In addition, further analysis based on gender was performed to complement the data related to the B-PQ.

No formal power calculation was carried out since the study relied upon the availability of the students of the ‘Virgen de Altagracia’ Secondary School to participate and their legal guardians to give their consent.

## 3. Results

Out of the 46 students that were initially recruited for the study, 2 were not available and the final sample consisted of 44 participants. The legal guardians of these two adolescents failed to provide the informed consent and were therefore excluded.

Table 1 shows the sociodemographic characteristics of the sample according to the risk of psychosis of the study participants. The majority of participants were male (65.9%) and had a mean age of 15.4 years (SD: 0.9) while the percentage of females was lower (34.1%). One hundred percent of the sample lived in a rural environment, they all lived in a village and most of the participants lived with their parents (81.8%). Regarding the economic situation of the families, only 9.1% of the participants indicated that they had economic difficulties. The rest of the variables are described in the table.

**Figure 1:**
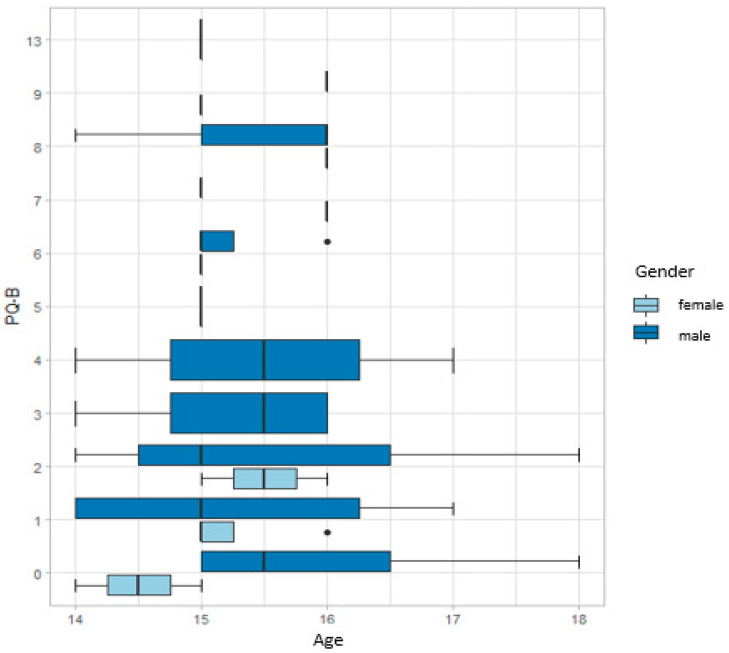
Results of the Prodromal Questionnaire Brief Version (PQ-B) test scores according to the age of the participants.

The results of the risk of psychosis can be seen in Figure 1. Overall, 38.6% of the participants are at risk of psychosis. The majority of the sample were male and, among them, the highest PQ-B response scores were found in the 15 to 16 year age range. The maximum score of all participants was 13. On the other hand, the interval under 15 years old corresponds to the lowest scores for the risk of psychosis (score 0).

In relation to gender, 70.6% of the male participants were at risk of developing a psychotic disorder. In contrast, the risk of the females was 29.4%. Is it is also interesting to highlight that the highest percentage of adolescents at risk was obtained among those participants lived with their parents (82.4% of them).

Regarding the explanatory factors of the risk of psychosis (Table 2), we observed that 75% of the population had a sedentary lifestyle, which is an important contributing factor. On the other hand, although the results show no significant differences in the contributing or protective factors, the majority of the participants at risk had high scores of the contributing factors, with 64.7% of the adolescents at risk consuming alcohol and 82.4% reporting stress. Only 6.82% occasionally consumed cannabis and 15.91% reported that they had been victims of bullying.

## 4. Discussion

Based on the results of the study, the data obtained provide a first insight into the psychosis risk vulnerability in adolescents in Spain, who are exposed to daily risk factors that may trigger the onset of the disease. This is a preliminary study and has therefore been carried out with a small sample size. Probably due to the sample size, the data are not significant but show preliminary evidence that analyses the risk of psychosis in adolescents aged 14–18 years. Overall, our findings indicated that more than one third (38.6%) of the sample studied were at risk of developing this type of disorder. The obtained data also suggest that alcohol consumption and stress were the most prevalent contributing factors in the participants. In addition, physical activity as a protective factor for mental health was present in more than half of the sample.

The mean age of the sample was 15.38 years. This age corresponds to adolescence, a stage of the life cycle in which, according to the scientific evidence, substance consumption can limit the potential of adolescents and can have a negative influence on their mental health [16,38,39]. In fact, the WHO-supported multicentre cross-sectional study carried out in 18 countries [40] identified adolescence as a vulnerable stage and a focus of risk for the development of these disorders. Our data also showed that 70.6% of males are at risk. This coincides with the theory that the male sex is more vulnerable to the development of mental disorders [10,16,28,40]. Another influential sociodemographic characteristic is the economic level. The latest research [41,42] determines that a low economic level is a factor that has an impact on vulnerability. This is reflected in our results, as we could observe that the highest percentage of risk is found in adolescents whose families have a medium–low economic level. It also important to highlight that the place of residence of all the study participants was in the rural area, which could make their access to resources and mental health professionals more difficult if they finally developed a psychotic disorder.

The evidence indicates that in adolescence, habits and routines that can damage or strengthen mental health are acquired and they can affect the development and performance of adolescents [38,39]. In our study sample, habits that are not beneficial for mental health were identified. We observed that 65.9% of the participants consumed alcohol and 11.8% of the population at risk consumed cannabis. According to one of the latest meta-analyses available in the literature [14], cannabis is one of the most frequently consumed psychoactive substances in the general population. In this meta-analysis [14], the authors also highlighted that most of the subjects started consuming cannabis during adolescence and the fact that there is a direct association between substance consumption and the development of psychotic disorders. These results have also been reported by other authors in recent studies [11,12]. Our results also show that 82.4% of the adolescents were under stress. This can be considered as an alarming percentage, since the latest research concludes that stress constitutes one of the environmental factors associated with a higher vulnerability to developing psychosis [33] and is a predictive factor of the disorder as it destabilises the physiological dynamics of the cortex [13,14,15,43,44]. Therefore, stress management strategies may constitute an important component of the preventive interventions [45]. However, the individuality of the maturational process for each person, and the influence of particular experiences and social situations make it difficult to compare stress-related results with other types of studies [46,47]. As our results show a high percentage of the contributing factors in the adolescents, especially stress, we consider it necessary to continue in this line of research. Further analyses of these contributing factors and the possible causal relations between them would be important to address in future studies. Since psychotic symptoms are relatively common in young people [48], studies are needed to perform further research into this subject and allow us to develop early and effective therapeutic intervention approaches [49].

In contrast, our study sample also adopted healthy habits for mental health, as 59.1% of the adolescent indicated that the performed physical activity throughout the week. Physical activity constitutes a protective factor, as evidenced by Stubbs et al. [50], who carried out a study in 47 countries and showed that low levels of physical activity correlate with high levels of psychosis risk. In addition, it is interesting to highlight that the authors focus their findings on the regulatory power of the cerebral cortex [18].

The results of our study show that 38.5% of the adolescents surveyed are at risk of psychosis. This figure is higher than those reported in other studies carried out on adolescents enrolled in secondary schools, in which the authors found a risk of psychosis of 20.6% in Asia [51], 23% in Canada [52] and 28.5% in India [53]. They all concluded that this is a health problem that affects the world’s population, as the prevalence of psychosis is high and it is showing an increasing trend [14,51,52,53]. However, although our findings suggest that more than a third of participants are at risk of psychosis, it is possible that not all the studied population at risk will finally develop a psychotic disorder. Nevertheless, the figures may reach the 12% established by the Extremadura Disability Care Centre (CADEX) this year based on the cases of people with psychotic disorders assessed and treated by mental health teams in Extremadura [54]. Therefore, it can be considered that our data are close to the reality currently studied and evidenced, although due to the lack of statistical power, we cannot affirm a direct correlation in our study sample with one or several contributing factor(s).

### 4.1. Clinical Implications and Recommendations

The effectiveness of early detection and intervention programmes has been evidenced in the scientific literature [2,55] and their establishment and implementation is essential for the management and prevention of psychotic disorders. To date, the available research has found occupational therapy to be highly effective as a preventive approach in adolescents at risk of psychosis [38,39]. In particular, comprehensive group and family programmes [56], programmes focusing on negative symptoms (anhedonia, lack of energy, allodynia, unsociability) [57] as well as multidisciplinary programmes based on cognitive-behavioural therapy, in which the occupational therapist was part of the team [58,59,60] have been carried out. Although the success rate of these programmes was excellent, and even if their effectiveness has been demonstrated, they are hardly being implemented. In fact, all the authors of these studies make reference to the existing knowledge gap, as well as the need for research. The fact that these programmes are not being fully implemented is also supported by the results presented by the World Health Organisation (WHO) in the report “Investing in Public Health” [61]. In this report, the authors state that “there is a large imbalance between the need for care for mental disorders and the resources available”. In relation to early detection, no research has been found that analyses the risk of developing a psychotic disorder in Spain. This is why this work can be considered novel and pioneering in our country.

Delaying the initiation of treatment once the symptoms of a psychotic disorder have developed leads to a poorer prognosis [16], which also supports the need for early detection and intervention programmes. In adolescents, professional early detection of situations that can increase the risk of developing a psychotic disorder is vital. Adolescents, in addition to being immersed in a great deal of change that makes them vulnerable, are conditioned by socialisation agents such as family, friends and their environments. It is at this moment, when they begin to acquire responsibilities, building habits, routines and roles, that adolescents begin to be ‘authors of their own lives’, as Kielhofner [62] stated. New habits are no longer mainly family-directed and are often more guided and influenced by the social group to which they belong [2,20]. In this sense, occupational therapists would have an important role as socio-health professionals assessing the adolescent as a whole [14]. In fact, the latest update of the Occupational Therapy Practice Framework of the American Association of Occupational Therapy (AOTA) highlights the importance of the preventive role of the discipline itself [63]. Therefore, the intervention of the occupational therapist would emphasise the occupational areas that are developed during adolescence and are most vulnerable to imbalance at this stage. The most important are work, education, basic activities of daily living, leisure and spare time, as well as their execution patterns (roles, routines, habits) [45,46,47,48,49,50,51,52,53,54,55,56,57,58,62].

### 4.2. Limitations of the Study

There are some limitations to this study. The data collection was performed through self-administered questionnaires. Therefore, there could be a non-differential classification bias which could also contribute to an overestimation of the findings. In relation to this, because our data analysis is cross-sectional, it does not allow us to establish a causal link between the contributing factors and the risk of psychosis. Thus, we consider that it would be of interest in future studies to include more specific statistical regressions. Another aspect to consider is the fact that the totality of our sample comes from a rural environment, which makes it difficult to extrapolate our results to the general adolescent population.

The size of the sample was another limitation. However, there are no previous studies available in the literature and therefore the participation rate can be considered appropriate. This is a pilot study and is the first exploration and screening of the risk of vulnerability of adolescents to develop a psychotic disorder. The preliminary results obtained have provided us with the first step to reflect on the preventive role of occupational therapy. However, this study is a starting point for future research to be carried out with a larger sample and to be able to extrapolate the results to a national and international level, where it would be interesting to include the preventive role of the occupational therapist in primary care teams as well as in secondary schools. As our results describe exogenous risk factors, it would also be interesting in future research to correlate these data with results related to the endogenous factors of the adolescents (genetic factors, family history, personality and behavioural development, current diseases, etc.) and with the impact of neurodevelopmental and cortical activity on mental health during adolescence.

## 5. Conclusions

In conclusion, based on the results of this pilot study, the vulnerability and risk of developing a psychotic disorder in the sample of adolescents aged 14–18 years that was studied, is high. The contributing risk factors that showed high values in the studied population were alcohol consumption and the presence of stress. Physical activity as a protective factor was found in almost all the population studied. Early detection and intervention in the most prodromal stages of these mental disorders is vital for a better prognosis.

## Figures and Tables

**Table 1:** Sociodemographic characteristics according to the risk of psychosis of the study participants.

Sociodemographic Characteristics	*n*(%)	Risk of Psychosis ^1^	
Participants	Total	At risk	Not at risk
	44(100)	17(38.6)	27(61.4)
Age, mean (SD)	15.4 (0.9)	15.41	15.37
Gender			
Female	15(34.1)	29.4	37.0
Male	29(65.9)	70.6	63.0
Place of origin			
Village	44(100)	100	100
City	0	0	0
Cohabiting members of the family			
Mother and father	36(81.8)	82.4	81.5
Mother	5(11.4)	11.8	11.1
Grandparents	3(6.82)	5.9	7.4
Economic level of the family			
High level	2(4.6)	0.0	7.4
Medium level	38(86.4)	82.4	88.9
Low level or economic difficulties	4(9.1)	17.6	3.7

SD: standard deviation. ^1^ The risk of psychosis was established according to the limits established by the scores of the Prodromal Questionnaire, Brief Version test norms. (PQ-B).

**Table 2:** Comparison of the percentage prevalence of the participants at risk of psychosis according to the explanatory factors.

		PQ-B ^1^	
	Total (*n* = 44)	At Risk (*n* = 17)	Not at Risk (*n* = 27)
	*n*(%)	%	%
Sedentary lifestyle (time spent sitting)			
8 h a day or less	33(75.0)	64.7	81.5
More than 8 h a day	11(25.0)	35.3	18.5
*p*-value ^2^	0.2887		
Alcohol consumption			
Occasional (weekends, at parties)	29(65.9)	64.7	66.7
Never	15(34.1)	35.3	33.3
*p*-value ^2^	1		
Cannabis consumption			
Occasional (weekends. at parties)	3(6.8)	11.8	3.7
Never	41(93.2)	88.2	96.3
*p*-value ^2^	0.5495		
Academic stress			
Yes	34(77.3)	82.4	74.1
No	10(22.7)	17.6	25.9
*p*-value ^2^	0.7161		
Victim of bullying			
Yes	7(15.9)	11.8	18.5
Accomplice	14(31.8)	23.5	37
No	23(52.3)	64.7	44.4
*p*-value ^3^	0.4239		
Traumatic event			
Yes	22(50.0)	52.9	44.4
No	22(50.0)	47.1	3.7
*p*-value ^2^	0.8525		
Family history of psychosis			
Yes	6(13.6)	17.6	11.1
No	38 (86.4)	82.4	88.9
*p*-value ^2^	0.6619		
Physical activity			
Never	9(20.5)	23.5	18.5
2 or 3 days a week	26(59.1)	70.6	51.9
Every day of the week	9(20.5)	5.9	29.6
*p*-value ^3^	0.1633		

^1^ The risk classification of psychosis extracted from PQ-B was established according to the cut-off points described by the authors (No risk: <6; At risk >or = 6); ^2^ *p*-value calculated with the Fisher’s Exact test; ^3^ *p*-value calculated with the Chi-Square test.

## Data Availability

The data underlying this article cannot be shared publicly to maintain the privacy of individuals that participated in the study. The data will be shared on reasonable request to the corresponding author.
